# Who Cares About Young People? An Ethical Reflection on the Losses Suffered by Adolescents, Beyond Those of School and Education, During the COVID-19 Pandemic

**DOI:** 10.1007/s11217-022-09860-6

**Published:** 2023-01-03

**Authors:** Gottfried Schweiger

**Affiliations:** University of Salzburg, Salzburg, Austria

**Keywords:** Adolescence, COVID-19 Pandemic, Good Life, Ethics

## Abstract

Adolescence is a valuable phase of life, not just because it is the phase of learning in school and preparing for a working life. During the COVID-19 pandemic it became clear that the rights, experiences, and lifeworlds of adolescents are considered less important than the needs of school, work, and productivity. However, there is an ethical claim for people to have a good adolescence, and this means that the losses of social contact, experiences, time, and space demanded of adolescents, in order to protect older and vulnerable groups during the COVID-19 pandemic, should be taken seriously. A distinctive quality of adolescence is that it cannot be repeated, nor can these experiences be replicated as adults. First experiences of independence, friendship, love, informality, recklessness, and youthful exuberance are intrinsically valuable and cannot be substituted for later in life. It is therefore not surprising that adolescents have sought and found their own spaces during the pandemic, some of them forbidden, because lock downs and closed social spaces have relegated them to the confines of their childhood bedrooms. In this paper, I explore an ethic of a good adolescence, which was impacted on by the COVID-19 pandemic, and consider what can be learned from this situation. Adolescents are to be taken seriously, their experiences are no less valuable than those of adults, and the losses they have suffered for the benefit of others should be honored. As far as possible, young people should be supported to have a voice in public discourse and in finding spaces to be adolescents.

## Introduction

The COVID-19 pandemic has been the defining event of recent years. It has drastically changed the lives of almost everyone. In addition to the medical challenges it presents, social problems caused or exacerbated by the pandemic quickly emerged. The pandemic is also a social problem. Social position and socioeconomic status have a major impact on who was able to protect themselves from infection and how successfully they could do this, on who was vulnerable, and on who was affected by the measures taken against the pandemic (for example, curfews, closure of stores and schools) and to what extent they were affected (Paremoer et al. 2021). Having a secure job, a spacious house with a yard, a functioning social network, financial resources, all these things make a critical difference in getting through a pandemic well, through a week-long lockdown, through an economic crisis, through periods of closed public spaces. But the pandemic not only has a social gradient, but also one based on age. Early on, it was clear that older people were particularly at risk of serious illness and death. Meanwhile, it is also clear that the social measures to fight the pandemic affected all age groups and that the pandemic has taken a heavy psychological toll, in particular affecting adolescents and children. This discussion is not intended to start a debate about offsetting or a comparison of different victim groups or losses due to the pandemic. Such offsets are certainly necessary, for example to assess and legitimize the cost-benefits and risks of certain measures. The protection of the lives of elderly and vulnerable people, the protection of critical infrastructure, especially medical facilities, justifies far-reaching measures and restrictions on individual freedoms. Nonetheless there are great social, political, scientific and, above all, ethical dilemmas here. Young people are, fortunately, only very rarely affected by the severe illnesses caused by COVID-19 and, compared to older people, only very few have died from COVID-19. Nevertheless, the pandemic has also led to massive losses in well-being and flourishing among young people. These losses deserve to be acknowledged; not only must they be included in an analysis of decision-making during the pandemic, but above all there is a need for societal and political attention to these problems and losses in young people. It is an ethical requirement to take young people’s concerns and needs seriously, especially when trying to decide whether or not severe curtailments of young people’s lives, harsh restrictions on their freedom, and great losses in education, mental health, and well-being are justified and necessary in order to protect older and vulnerable groups from infection. This attention is needed in the larger discourse of society, politics, and the media, but also appropriate attention is required in the small discourses that take place in communities, families, schools, and neighborhoods.

This essay addresses the situation of young people who have experienced great losses over the last two years, due to the pandemic, young people whose concerns were only belatedly and timidly noticed by society and politics. This finding is also valid when it is realized that schools came into the focus of pandemic management early on. After a chaotic phase in the spring of 2020, in which schools were suddenly closed in almost all of Europe, there was a normalization in school operations, but in many countries, such as Austria, this was repeatedly broken by the pandemic, by further lockdowns and by mandatory distance learning. School is a central place for young people, both as a physical geographic location and building and as a social site of interaction, learning, and conflict. For policymakers, two issues were at the forefront: first, the fear that educational loss would occur, which it did (Engzell et al. 2021). Second, the recognition that schools are important to relieve the burden on parents, i.e., to allow them to continue to participate in the workforce. Both aspects tend to express a view of childhood and adolescence that focuses on their role as adults-to-be and for adults and the adult world. Education is understood - this trend has been observed for some time, independently of the pandemic—as mainly instrumentally valuable, as training to be economically successful afterwards. The importance of education in all its facets as social, psychological and knowledge formation for a good childhood and adolescence, on the other hand, recedes into the background. Children have a right to education, but not only because it is economically useful to have educated adults, but also so that they can grow personally, their talents can flourish and they can live their lives according to their own ideas.

The reproduction of social inequalities through the education system—in some countries like the U.S. intertwined with racial-based inequalities - is thereby mostly accepted or promoted through certain policies of unequal provision of schools, privatization, and lack of support for disadvantaged children and young people. Such social inequalities in and through the education system were exacerbated by the pandemic (Darmody et al. 2021). The approach of policymakers, however, has been predominantly to ensure education without much consideration of the social interests and needs of children and young people. Adolescents, however, are not just people in education going through school; they are people in a specific phase of life with their own needs and interests, with their own lifeworld that is not absorbed into that of adults. Young people are therefore also resistant to the subsumption of their interests under those of adults and their lifeworlds. Thus, the goal of this essay is not to reduce young people to their role as students, but to respect and recognize them as whole persons, with their own desires, concerns, needs, interests and ideas of what makes a good adolescence. In particular, this means asking young people what they actually have an ethical claim to, what makes their adolescence good and what makes it bad, and what influence the pandemic plays here. My perspective, then, is focused on young people, and does not see them merely as future adults who need to learn something in order to exit from an earlier stage of development as quickly as possible.

Of course, some limitations to the scope of my reflections are worth mentioning right away. There is no such thing as “one” adolescence, and young people are very different from one another. This already includes the fact that adolescence includes people with different age- and development-specific abilities, interests and needs. Adolescence is a socially constructed stage, meaning that the psycho-biological processes of change that underlie it are socially interpreted and affected as well. In some social strata, adolescents remain dependent on their parents for longer than others and become independent later, independence being considered a characteristic of adulthood. This prolongation of youth is also mirrored in some jurisdictions, where young people in their 20s can be treated as adolescents. On the other hand, there are young people who are left to their own devices at a very early age, whose life experiences are largely such that they lack a carefree adolescence. Particularly when making global comparisons, the differences between young people are enormous, but these can also be found within countries. There are vast differences between well-protected middle-class adolescents in the Global North and adolescents who live in the same country as them but who are homeless, or who have had early experiences of violence and trauma from attachment breakdowns, or adolescents who are institutionalized and no longer have family ties. It is not my goal to present a global narrative of adolescence during the pandemic. I also focus on typical experiences of adolescents in the Global North, particularly in Western Europe. Other differentiations that play a major role, such as those based on socioeconomic status, gender, migration experience, ethnicity or skin color, or illness and disability, I will likewise only ever be able to hint at without exploring each in the depth they deserve. My perspective is that of ethics and philosophy, which attempts to present a contemporary diagnosis of the losses of a good adolescence due to the pandemic and the measures taken to contain or mitigate those losses.

## Ethics of a Good Adolescence

In this section I outline an ethical concept of a good adolescence. This will then serve as a guide to describe and evaluate the losses and experiences of adolescents during the pandemic. The area of ethics is not an empirical one and not a purely conceptual-theoretical one, but it always consists of questions about what should be normative, what moral rights and duties exist and where have they been violated. Thus, without ethics, it is also not possible to evaluate the experiences of young people during the pandemic in terms of their moral content - without ethics, no normative moral judgments are possible at all. Such judgments, however, are necessary to make political trade-offs, which are especially urgent in a pandemic. Whether the protection of life justifies restricting civil liberties is ultimately also an ethical question that requires ethical judgments. The concept of a good adolescence is only one possible approach to analyzing the impact of the pandemic on young people from an ethical standpoint. An alternative to asking what makes an adolescence good would be to work out what moral rights and duties adolescents have and whether these were unjustifiably violated during the pandemic. However, there will be overlap between the two approaches: moral rights - or legal rights - refer to a concept of a good adolescence, that is, a set of normative moral beliefs about what makes adolescence good. When I speak here of the concept of a good adolescence, I do not mean a thick concept of the good that would prescribe in detail how adolescents should live so that their adolescence is considered good. It is, as I will show in more detail, a liberal idea of a good adolescence that gives great space to youthful autonomy, so that young people can realize their own ideas of the good life. My concept of a good adolescence must also not be understood in a subjectivist-hedonist way. Thus, a good adolescence is not fulfilled when adolescents are subjectively happy or satisfied. Rather, my ethical concept of a good adolescence aims at an objective determination. This also means that subjective satisfaction and the objectively good life do not coincide for some adolescents. Not all adolescents who feel happy have a good adolescence. There may be many reasons for this, such as the phenomenon of adaptive preferences or alienation from oneself. Humans are capable of feeling moments of happiness and contentment even in very adverse circumstances, for example, escaping into addictions and drugs can artificially create such moments. But this is not what I mean by a good adolescence.

I would now like to present four dimensions of a good adolescence: Autonomy, which is the ability to live the life one chooses for oneself; Authenticity, which is the ability to live a life that is consistent with one’s identity; Security, which is the ability to live a life in which one is protected from danger and risk; Relationship and Community, which is the ability to live the life one chooses to share with others. These four dimensions of a good adolescence assume that adolescence is a life phase of development where one is learning, growing, and trying things out. This seems to me to be central: Adolescents are not yet adults, but they are also no longer children. They are in a phase of life characterized by a dialectic of independence and dependence and the search for self with guidance from others (Graf and Schweiger 2017). So how do I arrive at these four dimensions of a good adolescence, how are they justified? It is beyond the scope of this paper to detail and justify the philosophical methodology of the concept of a good adolescence - or a good life in general. I am guided by groundwork done in particular by Gunter Graf and Gottfried Schweiger in advancing the capability approach for children and young people (Schweiger and Graf 2015). They argue that the relevant capabilities to which children have a moral right must satisfy at least three criteria: they must be objectively determinable, they must be typically realizable for children and adolescents, and they must be of great importance for well-being and flourishing. In doing so, they accept that well-being and flourishing contain anthropological dimensions as well as those that are context-sensitive according to the level of societal prosperity the child finds themselves in. The last criterion is relevant for an ethical concept of a good adolescence and the four dimensions I present here are ones that are also suitable for describing dimensions of children’s and adolescents’ well-being and flourishing. They are derived from ethical reflection, which must also refer to scientific knowledge about adolescents in order to have a foothold in reality. These reflections do not take place in a vacuum, but relate to insights from other moral theories. For example, as Axel Honneth has argued, autonomy and authenticity are central values in the intellectual history of modernity, both for the individual and for politics (Honneth 1996). The dimensions mentioned here do not describe a good adolescence completely or comprehensively, and it is questionable whether this conception of the good adolescence can ever be fully actualized. My concern is to have a foil that can be meaningfully applied to the pandemic and its impact on adolescence.

I also want to address right away the question of how normatively these four dimensions are to be understood. One might get the impression that I advocate a perfectionism that demands that adolescents fully realize these four dimensions in order for their lives to be considered good. Here I am favoring a political rather than an individual or virtue ethics interpretation of the concept of a good adolescence. The four dimensions of autonomy, authenticity, security, and relationships are not binary but gradual. They exist on a continuum that depends on both individual and social conditions, including the preferences of the adolescents themselves. Thus, it is by no means the case that all adolescents should or must strive to achieve a maximum of these four dimensions in their lives. Rather, and therefore the political interpretation is relevant, it is about what society and politics should provide to adolescents and how to foster adolescent development.

Unlike children, adolescents have sufficient capacity for autonomy to be authors of their own biographies. Andrew Franklin-Hall (Franklin-Hall 2013) has distinguished between local and global autonomy, the former meaning the ability to make autonomous decisions that have a small impact and generate little path dependence. This includes, for example, autonomous decisions that can be easily reversed, that are not very demanding, or that do not pose a large potential threat. Global autonomy, on the other hand, refers to the ability to be the author of one’s own biography, i.e., to choose a particular life that one considers good. Decisions of global autonomy have serious consequences, are more complex, and sometimes have a greater potential for danger. This distinction seems important in relation to a good adolescence. For the most part, the discussion of adolescent autonomy and adolescents’ rights to decide for themselves is made up of the twin criteria of decision-making capacity and protection of well-being (the best interests of the child). This is about trade-offs between enabling and protecting (Anderson and Claassen 2012). However, I understand good adolescence as referring primarily, on the one hand, to the authorship of one’s own biography as an adolescent, that is, the question of what kind of life an adolescent wants to live. On the other hand, the question then arises of whether adolescents are autonomous enough to develop and decide for themselves what their own idea of a good adolescence consists of. Thus, I would like to focus on the distinction between local and global autonomy, which Franklin Hall relates to the entire biography, in the phase of adolescence. Adolescents, I suggest, should be able to live the life they want to live, that is, in this respect, they should be able to exercise their global autonomy of authorship of their biography as adolescents. This approach does not yet give exact criteria for the decisions adolescents should be allowed to make and how these should be regulated in interaction with parents or through laws. The elaboration of such criteria is necessary but, in my view, can only be done in relation to specific questions and issues. The guideline, however, should be about how young people can be helped to form and reflect on their own ideas of their lives as young people and to shape their lives accordingly. The concept of a good adolescence is open to a whole range of life plans, which young people decide on and which they consider to be right. However, it should be the young people’s own decisions, which may only be restricted in the case of great risks or clearly identifiable dangers. These restrictions must not be arbitrary or favor a certain version of a good adolescence. Neither the state nor parents have a right to intervene in this way. It is the task of parents, teachers and many other people and institutions to help young people become autonomous. This also involves a process of emancipation from old cultural, social, political or economic norms and practices, which young people should not adopt unquestioningly, but rather reflect upon critically. They must develop their own values. The great challenges facing the world (climate change, poverty, exclusion, political division…) demand that young people should be all the more critical.

Authenticity is the second dimension of a good adolescence, and it is closely related to the dimension of autonomy. Global autonomy is about being able to claim to be the author of one’s own life, while authenticity is about the good adolescence that one chooses being congruent with one’s own values, beliefs and standards. One can understand this as the introduction of a perfectionist element into global autonomy, but above all I am concerned with a simplification of the content of what global autonomy and authorship of one’s own life mean. Two aspects are important to me here. The first aspect refers to the fact that authenticity does not simply consist of implementing one’s own will. One can also want to be *in*authentic, if being authentic would result in negative reactions or disadvantages. Second, authenticity - like autonomy - is not free of preconditions, nor is it easy to achieve. Ideally, one can distinguish between authenticity as discovery and as creation (Levy 2011). Authenticity as discovery emphasizes the process of finding oneself when who one really is lies hidden and is brought out by searching within oneself. Authenticity as creation, on the other hand, emphasizes the aspect of making oneself who one wants to be. According to this model, one does not search within oneself for a hidden, true self, but this true self must be manufactured by one’s self. One difficulty that arises for this model of creation is that negative feelings and characteristics are probably also part of the authentic self, as are uncontrollable aspects of one’s emotional world. Obviously, however, there is overlap between these two ideal types, and authenticity is both found and created. It is also very difficult to state exact criteria for whether a person is authentic. (This is an old problem in philosophy, which comes up again and again for example in the critique of alienation or adaptive preferences). The search for the true self and the construction of this self, according to one’s own ideas, is always embedded in the social lifeworld and interaction with others. Therefore, authenticity can be spoken of not only as an ethical ideal (Bauer 2017), but also as a social ideal. We need others in order to become authentic and we are mutually dependent on each other to be authentic. Bernard Williams therefore also sees a close interlocking of authenticity, sincerity, and truthfulness (Williams 2002). These psychological and social processes begin in childhood and in adolescence they become central. The search for one’s own and authentic self goes hand in hand with the search for the various roles one wants to (or should) assume in the world and the processing of expectations of and from others. For adolescents, authenticity is a high value; they want to find themselves as well as to be able to be authentic in interactions with others (especially in close social relationships such as friendships or with romantic partners) (Thomaes et al. 2017).

Security is the third dimension of a good adolescence. I choose the difficult-to-translate German term *Geborgenheit* here because it expresses a deep dimension that includes both the feeling of being at home and the trust of being in good hands and being loved by another person. Thus, *Geborgenheit* combines a deep social relationship with the sense of security and familiarity that this relationship promotes. Clemens Sedmak has argued that security is central to a successful parent-child relationship, but that it means more than just an emotional relationship of love or an instrumental relationship of caring for the child’s needs (Sedmak 2016). *Geborgenheit* expresses a deep attachment, that there is a place of retreat, both an emotional retreat and a physical retreat where one can be with others but also alone with oneself. During adolescence, for the most part, security is still closely linked to the parental home as the space where one lives. For the most part, adults have to create the spaces of security they seek for themselves - their own home, where they feel safe. For young people, security means being protected and accepted, but at the same time already looking for their own spaces of security and making them for themselves. Therefore, security is also closely connected with the authentic and autonomous creation of private spaces where one can be by oneself. Security means that someone is there when you need them, but that you can also be there by yourself when you feel the need. Security offers the space to be authentic, with one’s mistakes and worries, to be listened to and to receive encouragement - but each young person must explore for themselves who they want to enter into this intimate relationship with and which people and social spaces provide security. Security is not just found by chance but must be created autonomously and authentically by all the people involved and there can be more than just two people. Not all adolescents feel secure with their parents, at all times and with regard to all their concerns. These relationships require care and long-term stability. Security is also an important dimension of a good adolescence outside the family. It plays a role in close friendships and communities where someone feels secure, in the sense that they are accepted there as they are (Peets and Hodges 2018). However, security also has a public and political dimension, that is, whether young people feel well cared for, accepted, and heard in the society in which they live. This is not, of course, the same close security that is significant in such intimate relationships as people have with parents, friends, or romantic partners. Social and political security can be expressed in feeling at home, in feeling safe, and in perceiving a place - for example, the neighborhood, the community, the country - not just instrumentally, but in building a positive emotional relationship with that place. The politics of security show a closeness to approaches in communitarianism and certainly also a danger that through the security of some people, others are excluded who do not feel secure and do not feel at home. This danger is to be countered - to stay with the politics of adolescence – by making sure that all young people, with their differences in needs, interests and ways of life, feel equally accepted and embraced. Young people should be able to feel secure and know that they can rely on society being a place for them where they don’t feel under attack or at risk because of their identities or beliefs.

A good adolescence is found when a young person is in relationship and community with other people. How much community and what kinds of relationship are important to a young person will differ individually. However, a good adolescence cannot be enjoyed without key social relationships, such as relationships with those adults who care for, educate, and accompany that young person. In the vast majority of cases, these adults are the parents with whom they share their life and who also have legal responsibility for the young person. The role of parents is both to give space to the adolescent, to support them in their educational and reflective processes - in search of themselves - and to be critical companions, providing protection and security from risks and dangers. While the central concept for the relationship between parents and children is love (Liao 2006), for adolescent children respect is added. Respect refers to recognition as a human being with one’s own desires and ideas of the good life, as well as being an actor. Here, respect for the autonomy and authorship of one’s adolescence, as well as for authenticity as the true self and the self that the adolescent finds within him or herself and forms according to his or her ideas, plays a central role. However, the parent-child relationship is not the only important one for adolescents. On the one hand, they enter other close relationships-friendships or romantic partnerships (Collins et al. 2009) and on the other hand, adolescents already interact in a variety of ways with people who are not close to them. Friendships, in particular, have a prominent role in development and well-being (Bagwell and Bukowski 2018). Friendships are also the beginning of the move from being centered on family relationships, most of which children and adolescents have not chosen, to relationships that are voluntary and in which there are no clear social hierarchies or dependencies (unlike parent-child relationships). Unlike children, adolescents’ lives are not purely private, but public, since they are political subjects and citizens (even if often they are not yet allowed to vote). How adolescents act in social relationships, both close and distant, which of these relationships they seek and with whom they deepen, is also a result of their autonomy and authenticity, that is, who they are and want to be (Peets and Hodges 2018). Commitment to values and causes that are important to the young person, that can also be expressed politically-publicly, is just one dimension of many that adolescents are entering into.

## Being Young in Times of a Pandemic

Now I come to the situation of young people during the pandemic. Against the background of the concept of an ethics of a good adolescence I have just outlined, I want to focus on the aspects that negatively influenced the possibilities of young people living a good life according to their own ideas. This also means that I am moving away somewhat from what is mostly focused on, namely, on the one hand, the question of health effects - the risk and consequences of infection - and on the other hand, the question of school and educational losses. Of course, both also play a role in a good adolescence, but other dimensions are just as relevant. School is a central social location for young people. Thus, the losses from school closures are by no means just educational losses or lack of opportunities for learning in the classroom (Engzell et al. 2021). There has been a loss of the social relationships that are made possible in and through school. Relationships could not be formed because of the lack of space for interactions. As physical spaces - in addition to school, at times parks, youth clubs, pubs, sports clubs, etc. - closed, virtual spaces took on greater importance. Such places are important for maintaining close social relationships with friends, but also with parents, siblings, and other close relatives living elsewhere.

The conditions and resources to deal with these losses are unequally distributed among adolescents. This refers to internal as well as external resources. Social dimensions and infrastructures as enabling conditions for a good adolescence play a major role. Adolescents from families living in poverty, who have fewer financial resources and often less space at home, had a harder time getting through the pandemic well. Social and psychological resilience factors that cannot be directly derived from the social environment play a large role. A group that received little attention, but was hit particularly hard by the pandemic, was young people living on the streets (Gewirtz O’Brien et al. 2021). Another similarly affected group were those who left state care during the pandemic (Roberts et al. 2021). The transition to adulthood is difficult for care leavers even without a pandemic. Not all settings have sufficient accompaniment for this process, resulting in an abrupt end to care. Social control of young people in state care is another aspect that worsened their situation during the pandemic (Gabriel et al. 2021).

The COVID pandemic has resulted in a great deal of psychological and social stress for adolescents, although it should be noted that, despite this, many have come through the pandemic well so far (Racine et al. 2021). In addition to social factors, gender, and relationship with parents, experiences made during the pandemic, such as the loss of a loved one, play a particularly important role. Consistent with previous research, it has been shown that girls are more likely than boys to engage in internalizing behaviors, that is, they are more likely to experience psychological distress due to anxiety, sadness, depression, or self-harming behaviors (Forte et al. 2021; Hawes et al. 2021). Similarly, more girls use their food to control their emotions and behavior, thus tend to develop eating disorders such as bulimia and anorexia (Solmi et al. 2021).

From a wider perspective, the pandemic was a time when there were limited opportunities for a good adolescence. This affects all four dimensions of a good adolescence - autonomy, authenticity, security, and relationships. The temporary restriction of the possibility to meet friends due to the closure of schools, public spaces, youth clubs or pubs threw young people back into the confinement of their childhood bedrooms. As a result, many adolescents were no longer able to maintain the social relationships that were important to them. Relationships with siblings - if any - or parents cannot compensate for this, as friendships, romantic love affairs or even loose forms of acquaintanceship with other young people have their own value for young people’s well-being and development. Therefore, they could also have fewer autonomous and authentic experiences, that is, experiences that correspond to their own idea of a good life and in which they want to find themselves. For adults, two central modes of self-actualization can be identified in modern societies: gainful employment and private life. For young people, gainful employment falls away, and school is not comparable to it, since it is not concerned with constituting oneself as an independent citizen who pursues an activity voluntarily in order to find recognition in it and to earn a living. While school is the essential public place where young people regularly interact with others, it is not a place of cooperative division of labor and productivity, but primarily and essentially a place of education. Therefore, private life also occupies a central place for a good adolescence. Restrictions on private life therefore also affect young people particularly; their places of retreat are not their own in a special sense - they live with their parents or, in rare cases, in homes, with foster parents or shared apartments.

An important aspect of a good adolescence is *Geborgenheit*, which includes the dimension of support in the development of their authenticity and autonomy, as well as emotional security and trust. The pandemic brought with it many uncertainties that generated anxiety. This was unavoidable, as knowledge of the duration of the pandemic, the severity of the consequences, and the risks of infection, illness, hospitalization, or death were unknown at the outset. This affected adolescents and adults alike. Adolescents, however, rely more heavily than adults on adult security, a role most often performed by parents. Adolescents are more independent than children, but they are also, unlike young children, very capable of informing themselves about the pandemic and understanding that information. Adolescents were directly confronted with the pandemic, through school, as well as being informed as media users. However, because they are not yet adults, young people have an increased need for safety - security - which enables them to live carefree lives. The phase of adolescence should not yet be characterized by the burden of the responsibilities of adult life, but rather a phase of freedom, learning and trying things out. The pandemic not only took away the ease and the social and physical spaces to do that, but it also created stress, uncertainty, anxiety, and anger among the parents and other adults who were supposed to help young people through this experimental phase of life. This is certainly not to blame the parents, or the social workers or teachers who had to deal with the situation of the pandemic themselves and suffered as a result. But it is noteworthy that the social dislocations generated by the pandemic - including financial worries or unemployment - fell on families (Donker et al. 2021). Similarly, the overwhelming demands of many parents to suddenly organize home schooling in addition to home offices had a negative impact, and not just on learning. Pandemic stress affected learning, but it also created stressors between parents and children. Women, in particular, had a triple burden to bear during the pandemic, especially mothers (Power 2020; Clark et al. 2021). They had to shoulder employment, care work, and teaching (for their children in home schooling).

Public and political perceptions sometimes devalue adolescent lives - adolescence is not yet “real” life, it is not about “really” important things. This expresses a prejudice from the point of view of adults, who value particularly highly their own lifeworld and its characteristics, including gainful employment, performance, personal responsibility, and thus adults may devalue the young person’s lifeworld, in which these things still play a subordinate role. For me, this also explains why the restrictions on young people’s freedom of movement and social contacts were not sufficiently considered as we developed pandemic policies. Also, the media often painted negative pictures of adolescents, for example, a Finnish study demonstrated how young people were reduced to two roles in the media: forward-looking students and reckless partygoers (Martikainen and Sakki 2021). In contrast, adults were positioned and portrayed more positively and in serious roles (it should not be denied that, in addition to age, social milieus and socio-economic position were also relevant, and as the pandemic progressed, an escalation of media discourse can be observed in parallel with social trends). Such differences in public and media perception are not new, but negative portrayals serve to compound negative images related to, for example, the political activism of young people (Bergamnn and Ossewaarde 2020). Prejudices and stereotypes of age groups exist on all sides, but young people have less power and fewer opportunities to influence the perceptions others have of them (for example, the media often report on young people, but much less often talk to young people and even less often are young people themselves the authors of this media coverage).

That the COVID pandemic leads to severe psychological distress, reflected in various medical conditions, is a serious consequence. But even if there were no such clearly diagnosable psychological sequelae, the ethical finding would be no less important. Even those adolescents who have come through the pandemic well and psychologically stable have, to a large extent, suffered such losses of autonomy, authenticity, security and community with other adolescents as a result of it. Inner strength or the presence of resilience resources should not obscure the fact that the COVID pandemic was a time when having a good adolescence on one’s own terms was not possible in the way it had been before.

Losses of a good adolescence cannot be compensated for. This has moral significance. Of course, no one, not even an adult, can be given back the time of the pandemic. Nevertheless, adult life is typically a much longer period of time than adolescence, the individual stages of life in adulthood often last longer and the experiences are largely more uniform. This is not to paint a distorted picture that adolescence is short, fast, and exciting, while adult life is long, boring, and uniform (although some people certainly experience it that way). Nevertheless, for the adolescent phase of life, the duration of two years or even a few months feels significantly longer than for an adult. This is also reflected in subjective perception. Adolescents are conscious, autonomous and authentic persons only for a short time (indeed, they become and make themselves so). The phase of childhood before adolescence may have lasted a few years, but it is neither so present nor so strongly experienced as part of one’s personality, because this personality did not yet exist in its mature form. Adults, on the other hand, look back on many years of a life “of their own” in which they had experiences that they wanted to have as authentic autonomous people and that reinforced them in this. Of course, there were also experiences in the lives of almost all adults that could not be had during the pandemic, that are not repeatable and were drastic. Someone who lost his or her father or mother and could not say goodbye because of the strict rules in the hospital, unfortunately, cannot get a second chance here either. Life is full of such singular, significant moments and experiences that are not repeatable. However, the period of adolescence is not just a period of time, a phase of life like all others in which such moments and experiences occur. Adults experienced many losses during the pandemic. However, adults did not lose one phase of their lives. Their adult lives, which last several decades, remain largely intact. They can catch up on many things that were important to them or continue to do them during the pandemic. The adolescent phase of life, however, is much shorter and the experiences in it are formative.

Thus, in my view, the comparison between adolescents and adults is relevant in determining what impact the pandemic had on adolescents and their chances of having a good adolescence. Such a comparison emerges from two perspectives. The first perspective asks whether adolescents are different from adults and therefore had different losses. This “otherness” can be understood as both anthropological and socially constructed. I think both dimensions play a role. The phase of adolescence as a bio-psychological developmental phase is indeed anthropologically different from the phase of adult life. It is also associated with different horizons of expectation and experience, formative developments that cannot be made up or repeated. Of course, adolescence is also a strongly socially constructed and shaped phase of life, which also means that the anthropological dimension is always framed by social norms and practices. This, too, plays a large role for a good adolescence and the consideration of the pandemic. Adolescents have different social spaces, different legal rights, different freedoms, because they have a different social position in society. For this reason, it was also easier to restrict them. And it is also these social aspects of adolescence, specific socially normed behavior and expectations or cultural dimensions of adolescence that suffered during the pandemic. The second perspective does not aim at differences between adolescents and adults but asks how a good adolescence and a good adult life refer to each other and whether a good adolescence as a concept and benchmark can only be had as distinct from and in relation to ideas about a good adult life. This can produce both positive and negative evaluations. Adolescence as a space of freedom without stress, responsibility and work or adolescence as a phase of restriction, immaturity and lack of independence. Both always point to the perspective from adult life. I argue for trying to take the losses of adolescence during the pandemic seriously, both in their own right, that is, without comparison to adults, and recognizing that the impact of the pandemic on adolescence should be seen in relation to adults.

I want to reiterate here that even without the pandemic, the opportunities for good adolescence were highly unevenly distributed among young people according to their socioeconomic status and membership in classes, strata, and milieus. Many young people in disadvantaged circumstances had far fewer opportunities to live an autonomous, authentic, and secure adolescence even before the pandemic. This structural injustice, however, does not make the losses of good adolescence during the pandemic any less painful or any less ethically problematic. The loss of a good adolescence must first be established before we can then ask further what the causes were, whether these losses could have been avoided, and whether and how they could be compensated for.

The pandemic as such, as well as regardless of the strict measures taken to combat it, represents an exceptional situation that is certainly reflected in the lives and experiences of young people. Even without all the initial restrictions, young people’s social relationships would have been severely disrupted, either because many would have reduced their contacts for fear of infecting themselves or others, or because their psychological well-being would have suffered greatly as a result of worrying about themselves or others. In this sense, the pandemic is a tragic event that leads to losses that are regretted but could not have been prevented or changed in principle. However, if we now look back over the past two years of the pandemic and see that the measures taken to combat it had a significant impact on the fact that young people did not have the opportunity to live a good adolescence, then we can see that the pandemic was precisely not only a tragic event, a natural disaster, but also a social process. As such it was an event that was managed in certain ways.

A particularly important role in managing the pandemic in all dimensions of a good adolescence was the use of new technologies, especially social media and messenger services, to maintain social contacts (Salzano et al. 2021). Life became even more digitized during the pandemic. Young people used these new technologies to stay in touch, maintain relationships, but also to seek and accept offers of help. There are both opportunities and risks associated with this. The new technologies made it possible to maintain relationships with friends, family and others, even create new ones, and were thus an important resource for dealing with the pandemic and upholding important dimensions of a good adolescence. How well this worked certainly depended on several factors, including the relationships that existed before the pandemic, the availability of these technologies, personal preferences and support from others or monitoring by parents (Foulkes and Blakemore 2021; Feng and Tong 2022). For some adolescents, therefore, the pandemic changed their lives less than it did for others, and for others the reduction in “real life” contacts was even a relief. The digitalization push has utilized already existing potentials; even before the pandemic, the social life of adolescents was strongly influenced by these new technologies, which also changed the dimensions of a good adolescence compared to previous generations. Autonomy, authenticity, security and good social relationships are being transformed by digitalization and the internet in terms of how they can be lived and how people want those dimensions to be lived (Yau and Reich 2018). This points to the social construction of the realization of these four dimensions. Expressed positively, one could say that the pandemic as a restriction of physical spaces and contacts as well as a threat to health has not endangered the digital component of a good adolescence, but that it has been able to have a relieving effect. It is therefore all the more striking that, despite the digitalization of adolescent life, the pandemic had such a serious impact as an event in “real life”.

On the other hand, virtual and digital exchange is not a substitute for social relationships in “real life”, in particular, meeting in physical spaces, having physical contact as well as seeing, smelling, touching other people, and the greater complexity and variety of non-verbal communication (Mesch 2019) (philosophical phenomenology would cite the German term “*Leib*” here, which cannot be replaced digitally). Digital technologies also pose risks that were amplified by their increased use during the pandemic. Some adolescents need more support and education to use technology wisely and safely. For example, during the pandemic, unsurprisingly, there was an increase in sexting, the sharing of erotic content between adolescents, especially within partnerships (Maes and Vandenbosch 2022). Sexting is a controversial topic, and not just from a parental and political perspective, because it is fraught with the risk of loss of control over the content created. Since it would not be either practically feasible nor ethically justified to ban young people from sexting in general, appropriate support is needed to ensure that young people can engage in this practice safely and in a self-determined manner. Since romantic relationships are part of a good adolescence, insofar as they are freely and safely pursued by adolescents, the pandemic also had a negative impact on them. Digital technologies offered the possibility to stay in contact, also sexually, but the deficiency of these virtual relationships compared to physical intimacy is obvious.

## Conclusion: Overcoming the Adult Bias

If, then, there are good reasons to believe that the pandemic has led to a deterioration for many young people, thereby violating their claim to have a good adolescence, then it makes sense to ask how to deal with this assessment. It is not my aim here to make a calculation of the losses of adolescents versus those of adults. Such a calculation seems to me from the outset to be at risk of succumbing to the deceptive impression that a good life can be numerically captured so that an objective comparison of the lives of young people and adults can be made. This is already impossible because there is no position from which adolescent and adult life worlds could be compared with each other.

The more important conclusion, in my opinion, is that the pandemic allows us to re-evaluate the social value of a good adolescence. By this is meant that the losses of adolescents must be recognized as such. Only then will one do justice to their status as moral and political subjects and citizens. Young people also have the right to demand this recognition, just as they may and should demand recognition and consideration for their other important interests (e.g., regarding climate change). However, it must be remembered that young people are not yet adults and have a different social, political and economic status. It should not be imposed on them to stand up for their interests alone. From the perspective of a theory of justice that grants every member of society sufficient opportunities in life, it follows that there is a public responsibility for the welfare of young people. Greater consideration of adolescent interests in policy, not only but especially during a pandemic, and recognition that the past two years have produced irretrievable losses for many adolescents, should open the discourse on compensations and future opportunities. Since, as I said, lost adolescence cannot be made up for, and this loss is more severe than the loss of two years of life as an adult, it cannot be a matter of young people making up for their adolescence. Unfortunately, that is not possible. But it is possible to work through the pain of this loss on the one hand and to compensate for it on the other. The focus should not be on the pandemic alone, but on the situation of young people and their future prospects as a whole. Here, even if one looks only at the Global North, the picture is bleak.
